# Color as a gate to verification: Super-additive costs in oculomotor disengagement

**DOI:** 10.1167/jov.26.9.5

**Published:** 2026-09-03

**Authors:** Maximilian Stefani, Christina Saalwirth

**Affiliations:** 1 Institute of Psychology, Universität der Bundeswehr München, Neubiberg, Germany

**Keywords:** visual search, oculomotor disengagement, feature overlap, saccadic latency, priority map

## Abstract

Visual search theories distinguish between attentional capture and disengagement, yet how multiple feature dimensions combine to delay disengagement remains unclear. This study investigated whether shared features (color, shape, object category) produce additive or super-additive costs in oculomotor disengagement. Forty-three participants performed a delayed-disengagement task where a central fixated stimulus shared zero to three features with a peripheral target. Saccadic latency (SL) served as the index of disengagement cost. Results showed that SL increased nonlinearly with the number of shared features, following a quadratic trend. A component decomposition revealed that this acceleration was driven primarily by color: Color overlap produced large main effects, whereas shape and object-category overlap produced costs primarily when combined with color. Our findings suggest that disengagement is not a simple summation of feature similarities. Instead, color may act as a high-priority gate: A color match may open a capacity-limited verification stage in which each additional shared feature disproportionately prolongs inspection. We interpret these findings in terms of a candidate hierarchical gating account, in which high-salience features such as color may govern entry into verification.

## Introduction

When searching for a specific children’s book on a bookshelf, we typically rely on a clear mental representation of the target. We may search by color—especially if it is distinctive; by size/shape, if the book stands out physically; or semantically, by recalling its title (a top-down cue that is highly informative when books are alphabetized). Often, we combine such cues—a form of conjunction search in the sense that the target is defined by a combination of features (Treisman & Gelade, 1980). However, if the target’s features are not unique, search slows because attention is repeatedly drawn to similar objects (distractors).

This everyday observation captures a core principle of visual search: The efficiency of finding a target depends on how distinctly it can be prioritized relative to its surroundings, that is, how strongly it is weighted on the priority map (Wolfe, 2021). The present study asks how unique features shape search performance, with emphasis on the temporal dynamics of attentional selection. Specifically, we examine whether and how combinations of shared features—that is, distractor–target feature overlap across dimensions—produce additive versus super-additive costs.

When searching for a visual target among distractors, eye movements provide a direct behavioral window into attentional control. The latency of the first saccade away from a centrally fixated (start) stimulus is a sensitive index of how strongly attention is held by the fixated stimulus. Because gaze is anchored on the center stimulus at display onset, variability in this first-saccade latency primarily reflects the time required to reject the fixated stimulus and initiate disengagement. Recent work shows that oculomotor behavior reflects two processes: (a) the initial capture of attention by a distractor and (b) the subsequent disengagement from it (Stefani & Sauter, 2023; Stefani, Sauter, & Mack, 2020). Both can prolong the time until gaze reaches the target: Capture can misdirect the initial saccade, and delayed disengagement can postpone leaving a distractor once it is fixated. Even with complex, real-world stimuli (e.g., needles, spiders), delayed disengagement from to-be-ignored stimuli has been demonstrated (Saalwirth, Stefani, Sauter, & Mack, 2024), indicating that not only simple features but also higher-level properties can shape the time course of selection. This raises the question of how distinct feature dimensions jointly shape oculomotor delays during search. Accordingly, we test whether costs from shared features across dimensions are additive or whether they interact to produce disproportionate slowing, resulting in super-additive effects.

### Attentional capture in visual search: models and mechanisms

Classic theories—including feature integration theory (FIT; Treisman & Gelade, 1980) and guided search theory (GST; Wolfe, 2021)—propose that attention is guided by a combination of bottom-up salience and top-down target expectations. These frameworks posit a *priority map* that weighs stimuli by physical salience and their match to the observer’s goals. Stimuli that uniquely stand out (e.g., color singletons) attract attention in a stimulus-driven manner, while stimuli sharing defining target features gain priority via the observer’s attentional set (*contingent capture*; Folk, Remington, & Johnston, 1992). Logan’s CODE theory of visual attention likewise formalizes competition in terms of similarity/grouping weights (CTVA; Logan, 1996). These accounts successfully predict which stimulus is selected first (e.g., a salient or target-similar stimulus often wins initial competition). However, they largely underspecify how selection-related competition translates into time costs once a stimulus has won selection—namely, how long it is inspected, how quickly attention disengages, and how overlap across multiple features combines to shape fixation dwell and disengagement dynamics. In other words, classic frameworks explain oculomotor capture and assume verification at a conceptual level, but they do not provide explicit commitments about the temporal mechanics of leaving a nontarget.

More recent research highlights this additional control stage. Born, Kerzel, and Theeuwes (2011) dissociated capture from disengagement using an adapted oculomotor capture task: Sudden-onset distractors primarily drove initial capture (bottom-up), whereas disengagement duration depended on top-down similarity to the target (e.g., shared target color prolonged dwell). Thus, a distractor can both attract gaze and hold it, but for partly different reasons. Building on this, Stefani and Sauter (2023) combined capture trials with dedicated delayed-disengagement trials to quantify the relative contributions of the two mechanisms. They concluded that, on their task and stimuli, approximately two-thirds of the overall distractor-related delay was attributable to oculomotor capture and about one-third to disengagement—magnitudes that are task- and stimulus-dependent but underscore the necessity of modeling both processes.

This insight extends FIT/CTVA/GST beyond initial allocation to the *stay-versus-switch* decision once a distractor is fixated: A priority (or weight) map integrates bottom-up and top-down information to guide selection, but the temporal cost of leaving a nontarget depends on feature overlap with the target. What remains underspecified is which feature dimensions dominate these costs and how combinations of overlapping features scale the timing of selection.

Crucially, contemporary models like guided search suggest that visual selection operates in two distinct stages: a rapid, parallel “guidance” stage that assesses stimuli (often in the periphery) and a capacity-limited “verification” stage that requires foveal attention (Wolfe, 2021). In many paradigms, simple feature mismatches can be rejected during guidance, whereas stimuli that surpass a similarity threshold must be passed to verification for detailed inspection. In delayed-disengagement paradigms, the critical distractor is already foveated at display onset; here, the same-stage distinction maps onto a fast feature-level rejection at fixation versus a slower verification process before a saccade can be initiated. This implies that oculomotor disengagement is not a monolithic process but depends heavily on whether a fixated stimulus can be rejected via a rapid feature check or instead triggers time-consuming verification.

### Contributions to delayed disengagement

Pioneering work by Boot and Brockmole (2010) showed that when observers started on a center stimulus that matched the target’s color, they did not immediately leave it; the first saccade to the target was delayed. Subsequent studies (Blakely, Wright, Dehili, Boot, & Brockmole, 2012; Wright, Boot, & Jones, 2015) replicated that currently fixated, target-matching stimuli delay disengagement relative to unrelated stimuli (see also Stefani et al., 2020; Saalwirth et al., 2024).

In single-feature search, a color match reliably delays disengagement, whereas a color mismatch does not. In conjunction search (color + shape), color overlap usually produces larger delays than shape overlap, unless shape discriminability/salience is experimentally increased. Under those conditions, shape also delays disengagement but typically less than color (Hao, Zhang, Wang, & Sun, 2022). Early eye-movement work likewise established that shared features slow selection: First saccades are rapid/accurate with unique single-feature targets but slower and more error-prone under conjunctions where target and distractors share features (Findlay, 1997). The *distractor-ratio* effect shows that rare, target-like distractors disproportionately attract early saccades, speeding elimination of nontargets; when distractor types are balanced, guidance weakens and target-directed saccades slow, consistent with heavier verification demands and prolonged oculomotor disengagement when many stimuli partially match the template (Shen, Reingold, & Pomplun, 2000). Temporal dissociations further indicate that very early saccades reflect bottom-up salience, whereas later saccades—especially the second—are driven by top-down target similarity; when a fixated distractor shares target features, dwell increases before moving on (Siebold, Van Zoest, & Donk, 2011).

Identity-level similarity adds another layer. When a center stimulus (task-irrelevant stimulus) contains the same letter as the target, responses show benefits/costs—but crucially only when that stimulus also matches the target’s feature set (e.g., color), suggesting that identity is evaluated once a stimulus has passed a feature-based gate and is being inspected at fixation (Wright, Boot, & Brockmole, 2015). The same functional identity effect generalizes beyond letters and layouts: Identity matches speed, and mismatches slow responses given a feature match, demonstrating an identity-level component of the disengagement cost (Stefani et al., 2020). Object-category-level information can also guide attention and prolong inspection: Targets defined by alphanumeric object category elicit early selection signals, and object-category-matching nontargets attract attention alongside color/shape matches (Nako, Wu, & Eimer, 2014; Nako, Grubert, & Eimer, 2016). Moreover, object-based target templates—not single features alone—determine which stimuli are prioritized and dwelled on, indicating that identity-level representations structure guidance (Berggren & Eimer, 2018). Taken together, these findings imply that identity and object-category overlap can prolong verification and oculomotor disengagement and that such costs may be amplified when they co-occur with low-level overlap (color/shape), consistent with a gating account.

Crucially, object-category-based effects are not confined to abstract symbols. Using complex, real-world stimuli (e.g., spiders, needles), Saalwirth et al. (2024) showed that time to first fixation on the target increases when the initial fixation lands on a distractor from the same category as the target, relative to a categorically unrelated distractor. Similarly, Moores, Laiti, and Chelazzi (2003) showed that distractors semantically or categorically related to the target (e.g., a table when searching for a chair) were preferentially selected by the first saccade and slowed search overall—evidence that same-category stimuli are treated as plausible candidates and receive extended scrutiny before attention is released (see also Belke, Humphreys, Watson, Meyer, & Telling, 2008). Related work with photographs shows that object-category-matching distractors attract more fixations and slow search, especially when they are also visually similar (Alexander & Zelinsky, 2011). Although the broader literature reports mixed results regarding how consistently categories guide the *earliest* eye movements, a broadly convergent picture is that object-category information can guide and hold attention when object-category structure is visually coherent. Furthermore, object-category-based guidance interacts with template precision and object-category variability: When object-category members are visually consistent (low variability), observers can form precise object-category templates that guide attention efficiently toward object-category-consistent stimuli, but these same templates make it harder to distinguish targets from highly similar within-object-category distractors, lengthening verification times and target dwell durations when target–distractor similarity is high (Hout, Robbins, Godwin, Fitzsimmons, & Scarince, 2017; Robbins & Evdokimov, 2024). In naturalistic scenes, thematically related objects attract gaze even when task-irrelevant, and combined manipulations of perceptual and categorical similarity show that category matches reliably slow search when perceptual similarity is high (Seidl-Rathkopf, Turk-Browne, & Kastner, 2015; Yeh & Peelen, 2022). Thus, beyond color and shape, object-category membership itself can hold attention: When searching for a conifer, other trees (e.g., broadleaf trees) behave like strong, object-category-consistent distractors that attract gaze and delay disengagement, particularly when low-level features also resemble the target.

Across these lines of evidence, two working conclusions emerge. First, similarity is layered and cumulative: Color provides the earliest and often strongest guidance signal; shape contributes when discriminability is matched; object-category similarity prolongs verification and oculomotor disengagement primarily once low-level features match. Second, template fidelity can gate these costs, and it can do so in different directions depending on clutter and within-object-category similarity: Precise templates sharpen guidance, but in target-like clutter, they can increase verification time and dwell; conversely, coarse templates can weaken guidance and inflate the candidate set, which can also increase verification demands even if any single candidate is rejected more quickly. In sum, the time course of selection—initial capture and subsequent disengagement, indexed by first-saccade destination/latency and distractor dwell—is jointly determined by similarity across color, shape, and object category and by template precision; when multiple matches co-occur, their disengagement costs tend to compound. The present study focuses on quantifying these *disengagement* costs and testing whether they scale linearly across overlap or show interactions indicative of verification-related synergy.

### Aims of the study

Building on the theoretical and empirical basis above, the present study examines how distractor–target feature overlap affects *oculomotor disengagement* in visual search. We manipulate three feature dimensions—*color*, *shape*, and *object category* (category-level overlap; the three-feature condition constitutes a full target match)—each alone and in combination. Disengagement is indexed by saccadic latency (SL), the latency to initiate the first saccade away from the center stimulus. We treat increases in SL relative to the zero-overlap (neutral) baseline as *disengagement costs*. Because the critical stimulus is foveated at display onset, we refer to this procedure as a *delayed-disengagement* paradigm.

To make the logic of our predictions transparent, we consider feature overlap at two complementary levels. First, we quantify shared features as feature overlap, operationalized as the number of shared features (0–3), and ask whether SL increases linearly with overlap count or accelerates as overlap increases.

*Research Question 1 (RQ1): Nonlinear scaling by number of shared features*. We test whether increasing overlap delays disengagement linearly or nonlinearly. Based on two-stage models of search (Wolfe, 2021), we examine whether the increase in SL shows a convex pattern that can be approximated by a quadratic trend. Specifically, stimuli sharing zero or few features should often be rejected rapidly via a fast feature-level check (Stage 1). As overlap increases, distractors are more likely to cross a similarity threshold and enter a capacity-limited verification process (Stage 2). This stage transition implies that each additional matching feature becomes more consequential at higher overlap levels, producing a disproportionate increase in disengagement time—the feature-count manifestation of super-additive verification costs.

Second, we use a component (decomposition) model that codes each feature dimension as present/absent (C = color, S = shape, O = object category) and tests whether combinations of overlaps are *additive* (no interactions) or *super-additive* (positive interaction terms). Crucially, these two perspectives are linked: If overlap costs are purely additive across dimensions, increasing the number of shared features should yield an approximately *linear* increase in SL. By contrast, if multifeature matches trigger verification-related synergy (i.e., super-additive costs), the feature-count function should become *convex* (accelerating), because the marginal cost of adding another shared feature increases once the stimulus becomes a plausible target candidate.

*Research Question 2 (RQ2): Feature type and combinations (component model)*. In a complementary component perspective, we test whether overlap across dimensions produces costs that exceed the sum of single-dimension effects. Here, super-additivity corresponds to positive interaction terms (e.g., C × S, C × O, or O × C × S), indicating that the effect of one feature depends on the presence of another. Guided by the idea that multifeature matches are more likely to be treated as plausible target candidates and therefore prolong verification, we test whether SL increases disproportionately when overlaps co-occur (relative to the linear sum of single-feature overlaps). An additive pattern remains a viable alternative under a strictly independent-contribution view of feature weighting on the priority map.

In addition, we examine whether effects show a graded ordering and conditionality: *Color* exerts the strongest effect on disengagement (and early guidance), *shape* contributes a smaller effect—especially when its discriminability/salience is not boosted—and *category* adds costs primarily *conditional* on at least one low-level match (color or shape) (Hao et al., 2022; Wright et al., 2015; Stefani et al., 2020; Saalwirth et al., 2024). Concretely, we test whether color > shape in effect size, with category producing additional SL, particularly when gated by low-level overlap.

## Method

### Participants

The study included 43 participants (25 males, 18 females), all students of the University of the Bundeswehr Munich. In every case, biological sex matched gender. The mean age was 23.03 years (range: 20–28 years), and five participants were left-handed. All provided informed consent and received course credit per hour of participation if requested. All procedures adhered to the Declaration of Helsinki (1964) and were exempt from a full ethics review by the local ethics committee.

### A priori power analysis

Because prior delayed-disengagement work provides quantitative benchmarks primarily for single-feature overlap, our a priori power analysis was anchored to the most directly comparable and theoretically minimal effect: the within-subject contrast between a neutral center stimulus (zero-feature overlap) and a color-congruent center stimulus (one-feature overlap in color). In Stefani et al. (2020), color congruency produced a disengagement delay of approximately 31 ms (congruent vs. neutral). To remain conservative given our more complex, naturalistic stimuli and broader condition structure, we planned for substantially larger within-subject variability and assumed a standard deviation of the paired difference scores in the range of 45–55 ms. Under these assumptions, a two-sided paired-samples *t*-test power analysis (α = 0.05) indicates that detecting a 31-ms delay with 80% power requires approximately *n* = 19 participants (45 ms) to *n* = 27 participants (55 ms).

Notably, evidence from paradigms using complex pictures suggests that disengagement effects can be at least as large or larger than this conservative benchmark: In Saalwirth et al. (2024), saccadic latency differences between same-category and neutral stimulus conditions were on the order of 40–50 ms. This supports the assumption that powering the study for the 31-ms minimal effect constitutes a conservative lower bound for stimulus-based disengagement costs.

Our main questions extend beyond this minimal benchmark and address how disengagement costs scale across 0–3 shared features and whether multifeature overlap yields nonlinear (convex) scaling and super-additive interactions across dimensions. Because the existing literature does not provide directly comparable quantitative estimates for these multifeature patterns in the present paradigm, we did not base sample size on a specific quadratic term or interaction parameter. Instead, we powered the study for the conservative minimal effect that is well supported by prior work (color overlap vs. neutral). Multifeature effects and interactions were therefore treated as secondary and are evaluated primarily via effect sizes and confidence intervals rather than being assumed to be equally well powered a priori.

### Setup

Stimuli were presented on a 144 Hz LCD monitor (Eizo) at a viewing distance of 68 cm and a resolution of 1,920 × 1,080 px. Manual responses to stimulus orientation (left/right) were recorded via a Black Box ToolKit USB response pad (The Black Box ToolKit Ltd), with participants pressing the corresponding button using the left or right index finger. Saccadic latencies (in ms) were measured using an EyeLink 1000 Plus eye tracker (SR Research, Inc.) sampling at 1000 Hz.

### Task

**Figure 1.**
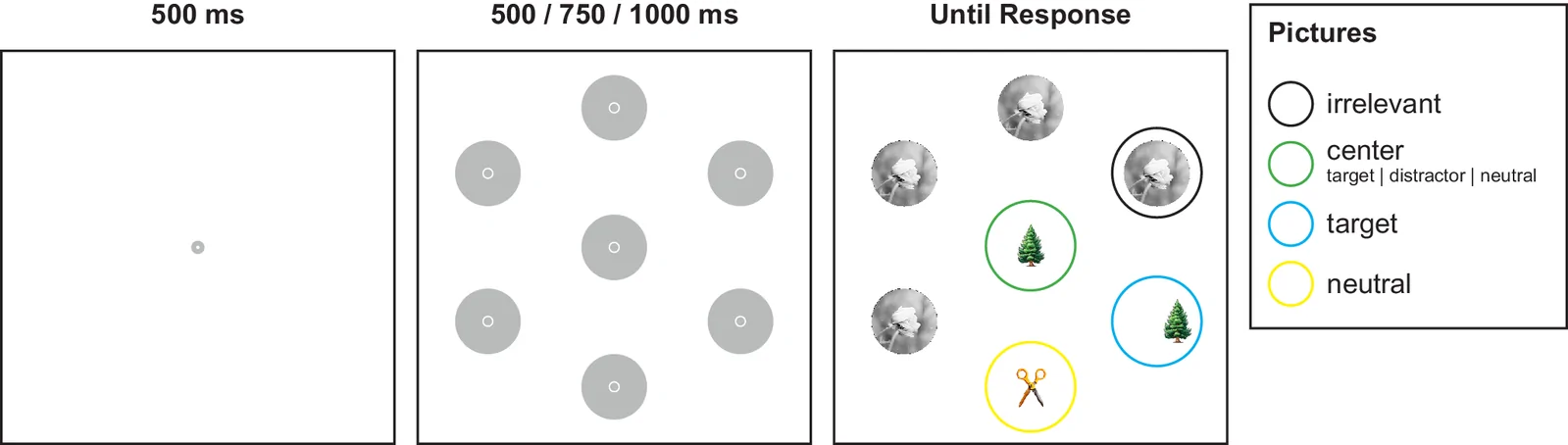
Example of the experimental setup. After a variable delay, gray circles were replaced with condition-specific stimuli. Participants fixated the center stimulus (green) until display onset, then searched for the peripheral target (blue). The center stimulus could be a target, a distractor (sharing one to three features), or a neutral stimulus (sharing none).

The visual search display consisted of six gray circles (RGB: 196, 196, 196; radius 1.7°) arranged around a center circle (radius 1.4°) at a visual angle of 7.8° on a black background. Each circle contained a smaller gray circle (radius 0.3°). Each trial began with a fixation point for 500 ms, followed by a variable delay of 500, 1,000, or 1,500 ms (randomized and counterbalanced). Subsequently, distinct stimuli (radius 1.7°) replaced all circles (see Figure 1). Locations of targets, distractors, and neutral stimuli, as well as stimulus orientations, were counterbalanced across trials.

**Figure 2.**
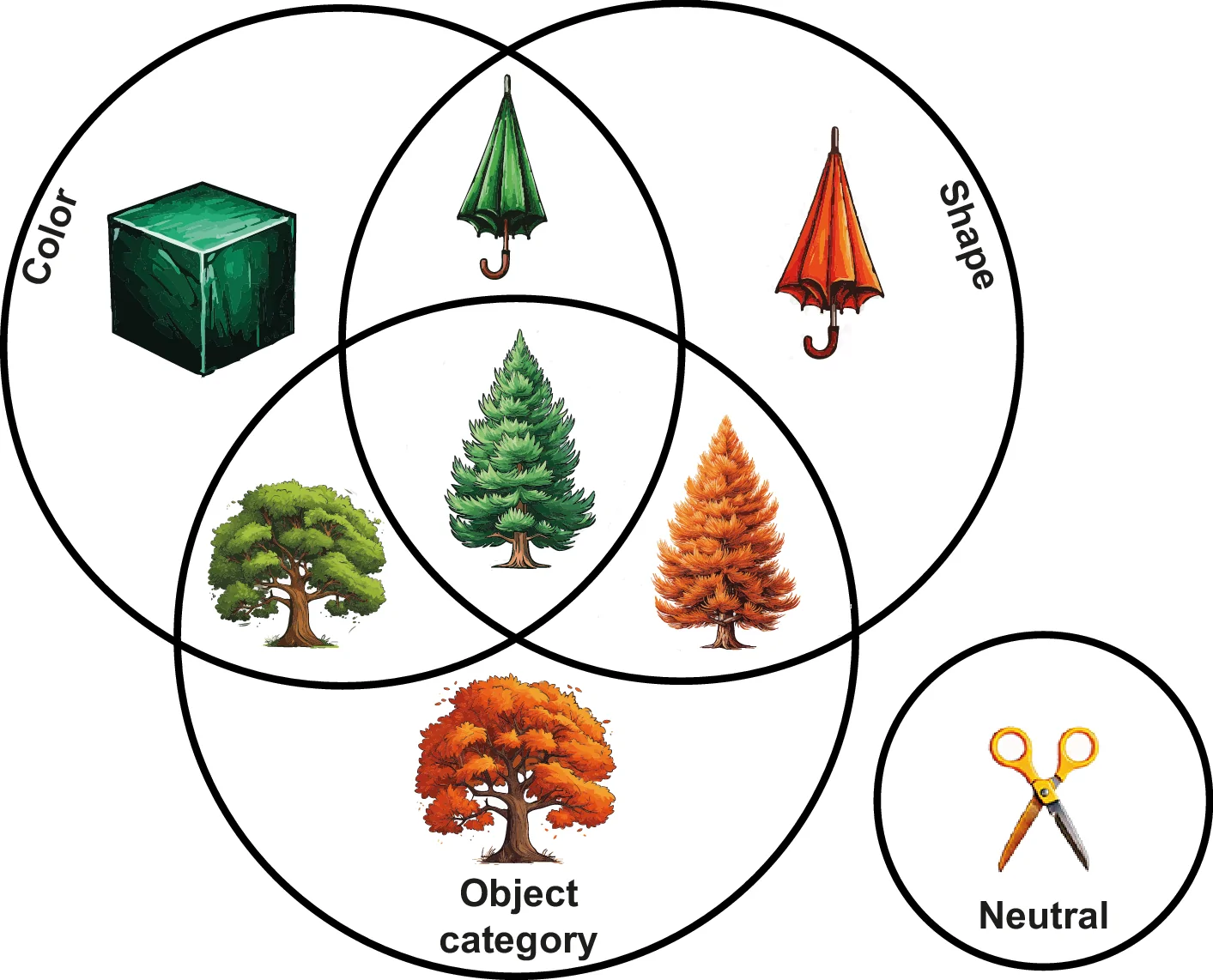
Example of shared feature conditions of one object-category set. This figure illustrates the distractor types presented in the center stimulus within each block. Distractors could share one of three feature dimensions with the target—color, shape, or object category—or they could overlap on multiple dimensions, as shown by the intersections of the sets. The target stimulus is depicted in the center; it defined the relevant feature values for each block and simultaneously served as the target in the outer search display. A neutral distractor, shown on the right, shared none of the target’s features and therefore served as a baseline with zero feature overlap. This object-category set establishes the full shared-feature structure used to manipulate distractor–target similarity across the experiment.

**Figure 3.**
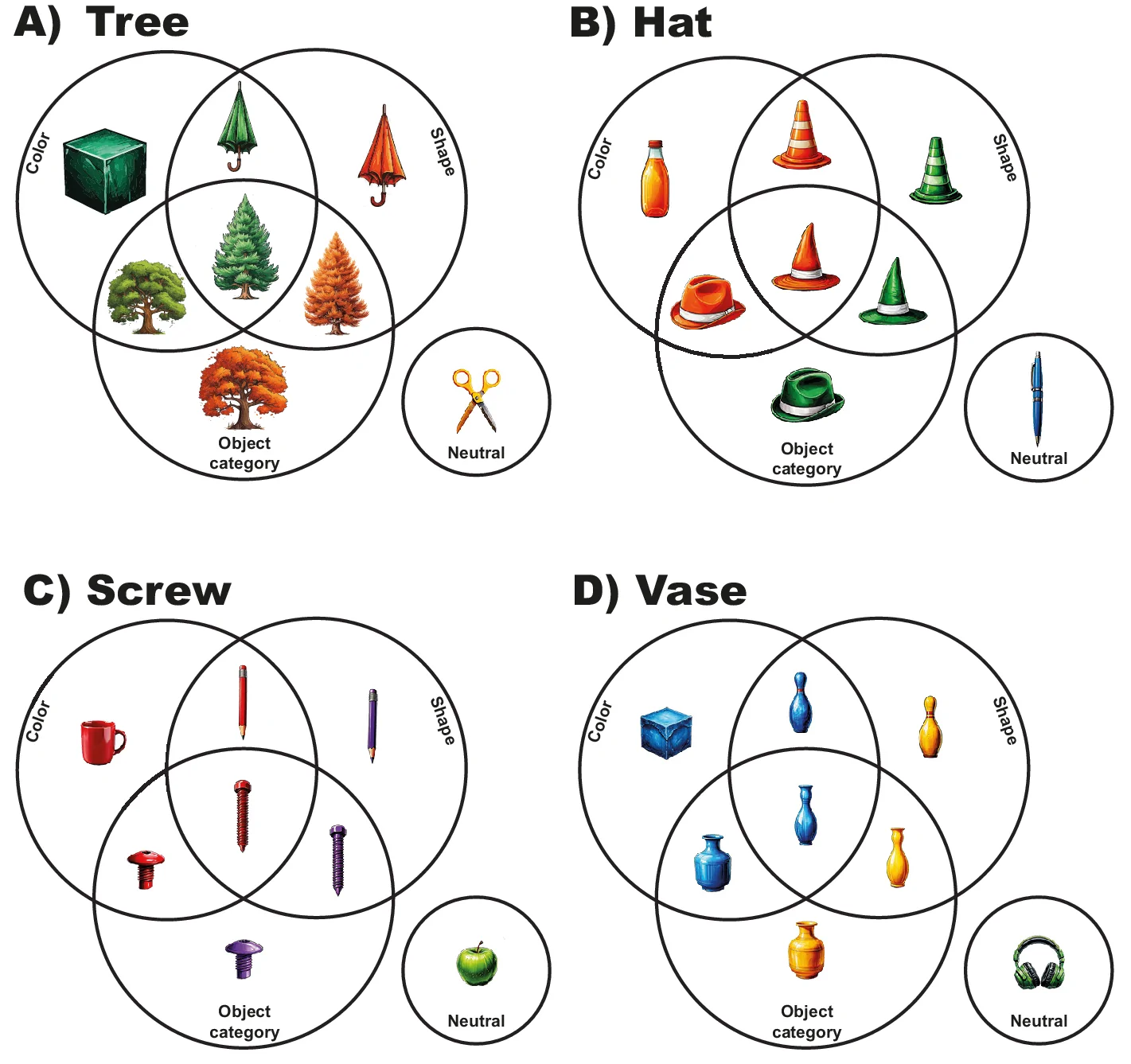
Shared feature conditions of all object-category sets. Shared feature conditions of all object-category sets. The targets for panels A–D are a tree, a hat, a screw, and a vase, respectively. Depiction of all object-category sets used across experimental blocks. In each block, these stimuli could appear at the center of the search display (center stimulus). The stimulus shown in the middle of each illustration (matching all three feature dimensions) served as the target for that block.

Participants were instructed to fixate the center stimulus until stimuli appeared and then to identify the peripheral target’s orientation (left/right) as quickly as possible. The center stimulus could represent a target (sharing three features: color, shape, object category), a distractor (sharing one or two features), or a neutral stimulus (sharing none; see Figure 2 or Figure 3 [all sets]). Four peripheral circles displayed irrelevant stimuli (grayscale flowers), one contained the target, and one contained the neutral stimulus. Each block used different sets of target, distractor, and neutral stimuli, repeated once (eight blocks total). The order of the four object-category sets across blocks was randomized separately for each participant, dissociating block order from category-specific difficulty and ensuring that practice and category were not systematically confounded. Each block comprised 64 trials; the first 10 of the first block were practice trials and excluded from analysis.

**Figure 4.**
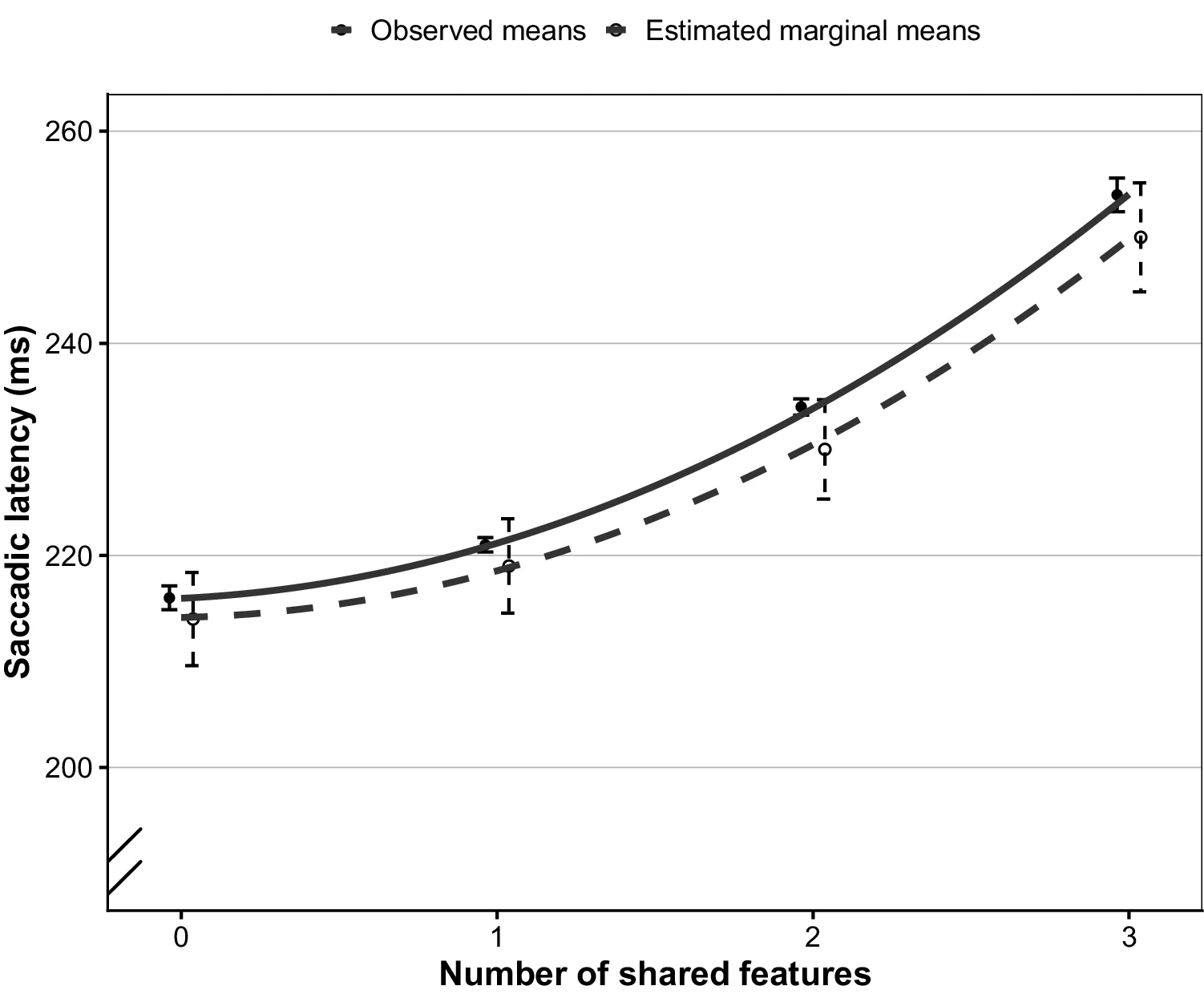
Quadratic trend of disengagement. Saccadic latency (in ms) was measured to quantify delayed disengagement from the center stimulus. The number of shared features between the distractor and the target was either 0 (neutral distractor), 1 (color, shape, or object category), 2 (any combination of two shared features), or 3 (the target itself). Filled circles show the observed mean saccadic latencies across participants and blocks. Open circles represent the estimated marginal means from the statistical model. Error bars indicate ±1 *SEM*.

**Figure 5.**
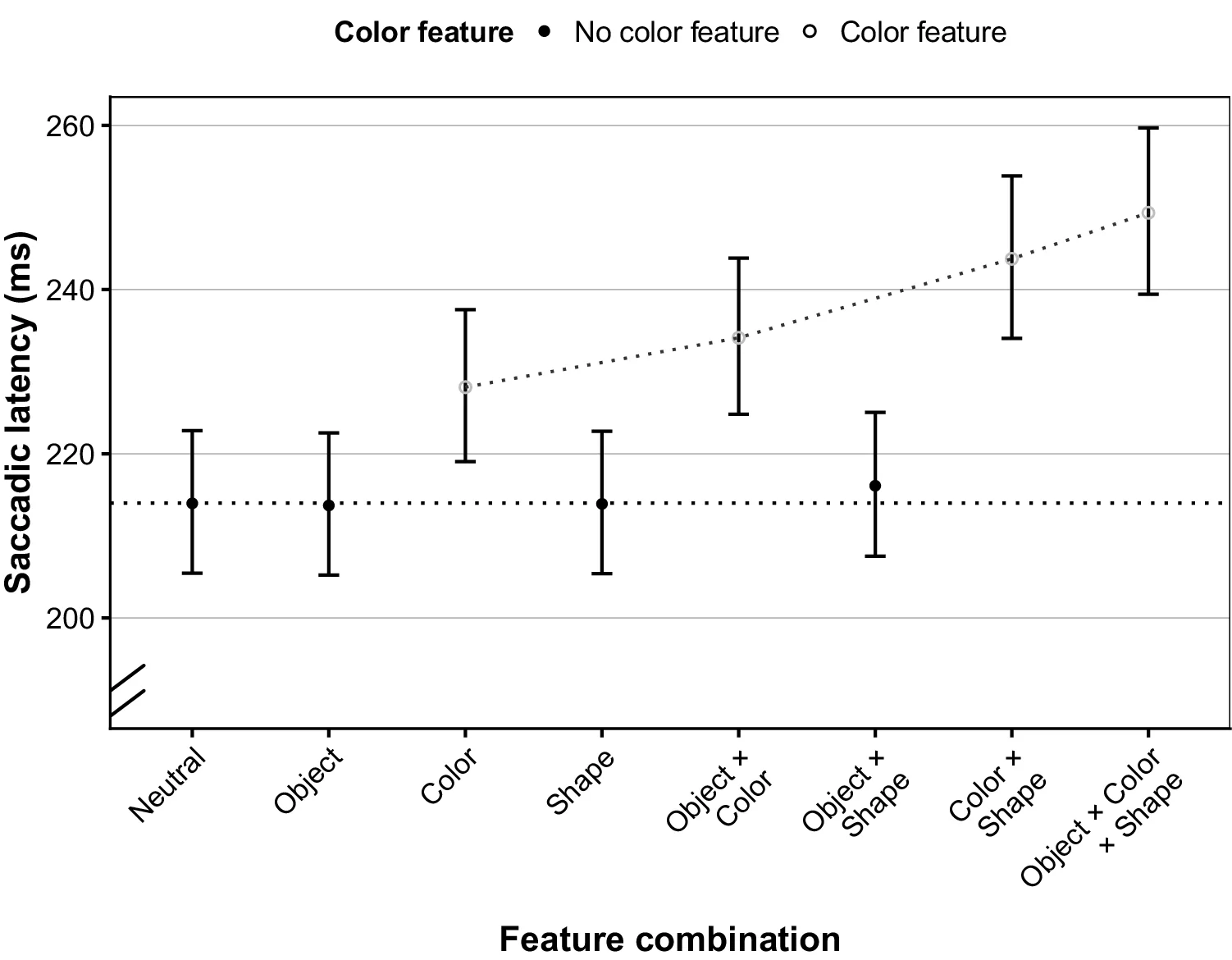
Component pattern of saccadic latency by shared features. Saccadic latency (in ms) as a function of the specific feature combinations shared between distractor and target. Axis labels denote which feature(s) of the target each center stimulus matched: Object (object category), Color, and Shape, together with their pairwise and three-way combinations; for example, Object × Color marks a stimulus sharing both object category and color, and Object × Color × Shape marks the full target match. These dimensions are abbreviated O, C, and S in the text and tables. Neutral distractors shared no feature with the target. Open circles denote conditions in which color matched the target; filled circles denote conditions without a color match. Error bars indicate ±1 *SEM*.

### Stimuli

All critical stimuli were AI-generated with Adobe Firefly to achieve a uniform visual style and tightly controlled similarity relations across conditions. Stimuli were organized into four *object-category sets*, each defining one target category: *hat*, *tree*, *vase*, and *screw*. Within each set, we created the full factorial overlap structure across three feature dimensions: *object category*, *color*, and *shape*. Feature overlap was implemented by design: Category-overlap stimuli were different exemplars of the same category with a different dominant hue and outline (e.g., a different hat or a different tree), color-overlap stimuli matched the target’s dominant hue while differing in category and outline (e.g., a nontarget stimulus in the target color), and shape-overlap stimuli were selected to approximate the target’s coarse silhouette while differing in category and hue (e.g., cone-like, tree-like, bottle-like, or elongated shapes). The neutral stimuli were chosen to differ from the target on all three dimensions. Targets were presented in two orientations (left vs. right), created by horizontal mirroring, and target orientation was counterbalanced across trials.

The three feature dimensions were not equated for perceptual discriminability, and object-category overlap was not fully orthogonal to low-level features: Within-category exemplars (e.g., different trees) inevitably shared some residual low-level properties—texture, internal color gradations, and silhouette subcomponents—beyond the manipulated hue and outline (see Discussion).

### Saccadic latency

Saccadic latency was defined as the time from search display onset to initiation of the first saccade leaving the center stimulus, which is defined as the area of interest (AOI, radius 2.5°). Decision time was defined as the interval between the first fixation on the target AOI and the corresponding manual response. Trials without target fixation were excluded. Eye movements were identified as saccades if they exceeded 0.2° and a velocity of 30°/s. The first saccade had to originate from the center stimulus (97.3% of all trials) and had to land outside the center (89.7%). Trials excluded by the latter criterion reflect small within-AOI eye movements that did not leave the center stimulus (e.g., overly short saccades or corrective movements following premature starts). Trials with latencies <90 ms (5.7%), blinks before saccade onset (0.8%), or incorrect responses (4.6%) were excluded. No participants were removed.

### Statistical analysis

To address the two research questions—whether a greater number of shared features between a center stimulus and a target stimulus leads to stronger delayed disengagement (i.e., longer saccadic latency) (RQ1) and whether this effect differs depending on the type of shared features (RQ2)—two complementary statistical approaches were employed.

To test RQ1, we first examined a graphical depiction of the relationship between SL and the number of shared features, which suggested a quadratic trend. In order to statistically test whether this relationship was best described by a linear or a quadratic function, two simple mixed-effects models were compared. In the linear model, the number of shared features was included as a single predictor of SL, whereas in the quadratic model, an additional squared term was added. Both models included random intercepts for participants. A likelihood ratio test was used to compare model fits. The test indicated that the model including the quadratic term provided a significantly better fit to the data. Therefore, in the next step, a quadratic mixed-effects model with covariates was calculated. In this model, log-transformed saccadic latency (to reduce skewness) served as the dependent variable. The number of shared features was entered as both a linear and a quadratic term to capture nonlinear effects. Block number and trial number were centered and included as covariates to control for practice and order effects. Object-category set was added as a categorical predictor and allowed the interaction with block number to account for potential differences in learning trajectories across blocks. In addition, random intercepts and by-object-category-set slopes were estimated for each participant to model individual differences in baseline performance and block-specific variability. Here is the resulting model equation:

$$\begin{array}{ccc} \;\log({\mathrm{SL}}_{ij}) & \;= & \beta_{0}+\beta_{1}\text{number}\;\text{of}\;\text{shared}\;{\text{features}}_{ij}\\ & & +\;\beta_{2}\text{number}\;\text{of}\;\text{shared}\;{\text{features}}_{ij}^{2}\\ & & +\;\beta_{3}\text{block}\;{\text{number}}_{c,ij}\\ & & +\;\beta_{4}\text{object-category}\;{\text{set}}_{ij}\\ & & +\;\beta_{5}\text{block}\;{\text{number}}_{c,ij}\text{object-category}\;{\text{set}}_{ij}\\ & & +\;\beta_{6}\text{trial}\;{\text{number}}_{c,ij}+u_{0j}\\ & & +\;u_{1j}\text{object-category}\;{\text{set}}_{ij}+{ɛ}_{ij}. \end{array}$$

To test RQ2, SL was analyzed using a component decomposition analysis (linear mixed-effects model). To reduce skew, SL was again log-transformed and served as the dependent variable. For the three feature components, new variables were created representing the three feature components—Object category (O), Color (C), and Shape (S)—each coded as binary (0 = feature not shared with the target, 1 = feature shared with the target). These variables (O, C, S) were entered as fixed effects in the model, including all interactions (C × O, C × S, O × S, and O × C × S). To control for practice and order effects, we added centered block number and centered trial number within blocks as covariates. Because practice trends can differ by object-category set (hat, tree, vase, and screw), centered block number was interacted with object-category set. The model further included a random intercept and random slopes for object-category set for each participant. Here is the resulting model equation:

$$\begin{array}{ccc} \;\log({\mathrm{SL}}_{ij}) & \;= & \beta_{0}+\beta_{1}O_{ij}+\beta_{2}C_{ij}+\beta_{3}S_{ij}+\beta_{4}O_{ij}C_{ij}\\ & & +\;\beta_{5}O_{ij}S_{ij}+\beta_{6}C_{ij}S_{ij}+\beta_{7}O_{ij}C_{ij}S_{ij}\\ & & +\;\beta_{8}\text{block}\;{\text{number}}_{c,ij}\\ & & +\;\beta_{9}\text{object-category}\;{\text{set}}_{ij}\\ & & +\;\beta_{10}\text{block}\;{\text{number}}_{c,ij}\text{object-category}\;{\text{set}}_{ij}\\ & & +\;\beta_{11}\text{trial}\;{\text{number}}_{c,ij}+u_{0j}\\ & & +\;u_{1j}\text{object-category}\;{\text{set}}_{ij}+{ɛ}_{ij}. \end{array}$$

All models were conducted in R (version 4.4.3; R Core Team, 2024) and RStudio (version 2025.09.2) using the packages lme4 (Bates, Mächler, Bolker, & Walker, 2015) for model estimation lmerTest (Kuznetsova, Brockhoff, & Christensen, 2017) for significance testing with Satterthwaite’s approximation of denominator degrees of freedom; emmeans (Lenth & Piaskowski, 2025) for estimated marginal means and Holm-corrected post hoc comparisons; effectsize (Ben-Shachar, Lüdecke, & Makowski, 2020) for reporting partial eta-squared ($\eta_{p}^{2}$) for each fixed effect, calculated from the Type III analysis of variance (ANOVA) of the mixed-effects models; and ggplot2 (Wickham, 2016) for creating all figures. Marginal and conditional *R*^2^ values were calculated following the MuMIn package (Bartoń, 2025). Models were fitted by restricted maximum likelihood (REML) with the bobyqa optimizer. Estimated marginal means (EMMs) were reported on the log scale and back-transformed to milliseconds (ms). Model assumptions (linearity on the log scale, homoscedasticity, and normality of residuals) were checked using standard residual diagnostics and showed no meaningful violations.

## Results

### Number of shared features

A graphical depiction of the relationship between SL and the number of shared features, which suggested a quadratic trend, can be found in Figure 4. Both models included log-transformed SL as the dependent variable and the number of shared features as the predictor, with random intercepts for participants. The quadratic model additionally included the squared term of the predictor, capturing a nonlinear trend. The model comparison (likelihood ratio test) indicated that the quadratic model fit the data significantly better than the linear model, χ^2^(1) = 78.19, *p* < 0.001. Therefore, a quadratic mixed-effects model including covariates was calculated next.

The quadratic mixed-effects model was fitted to further examine the covariates block number, object-category set, and trial number. Random intercepts and random slopes for object-category set were estimated for each participant. The results of the model can be found in Table 1.

**Table 1.** Type III ANOVA results for the quadratic mixed-effects model.

Effect	*df*_1_	*df*_2_	*F*	*p*	$\eta_{p}^{2}$
Number of shared features	1	17,168.9	3.88	0.049	<0.001
Number of shared features^2^	1	17,170.5	86.31	<0.001	0.005
Block number_*c*_	1	17,238.6	2.42	0.120	<0.001
Object-category set	3	38.1	3.31	0.030	0.207
Trial number_*c*_	1	17,177.2	20.82	<0.001	0.001
Block number_*c*_ × Object-category set	3	6,279.6	2.78	0.039	0.001

*Note:* Block number_*c*_ and trial number_*c*_ were mean-centered. Degrees of freedom are Satterthwaite approximations.

The analysis revealed a significant quadratic effect of the number of shared features. The linear term for number of features was significant but small, suggesting an initial increase that accelerated as more features were shared. Among the covariates, saccadic latency significantly decreased with trial number, reflecting within-block practice effects. There was a main effect for object-category set but no main effect for block number. The interaction between block number and object-category set was significant, suggesting that participants showed different learning trajectories across blocks depending on object-category set. The fixed effects explained 6.2% of the variance in log-transformed saccadic latency (marginal *R*^2^ = 0.09), whereas the full model including random effects explained 42.2% (conditional *R*^2^ = 0.45).

Estimated marginal means (back-transformed from the log scale) showed that the model-adjusted means for SL increased from zero to three shared features and support a quadratic trend (see Table 2). Pairwise comparisons across the number of shared features (0, 1, 2, and 3) indicated that all comparisons were statistically significant (see Table 3). Although the quadratic effect of number of shared features explained only a small proportion of the residual variance ($\eta_{p}^{2}$ = 0.005), the condition differences were substantial and practically meaningful, with small to large effect sizes Cohen’s |*d*| = 0.14 − 0.90.

**Table 2.** Estimated marginal means of saccadic latency by number of shared features.

Number of shared features	*M* (ms)	*SE*	95% CI_*LL*_	95% CI_*UL*_
0	214	4.39	205	222
1	219	4.45	210	228
2	230	4.69	221	240
3	250	5.14	240	260

*Note:* 95% CI_*LL*_ = lower limit of the 95% confidence interval; 95% CI_*UL*_ = upper limit of the 95% confidence interval.

**Table 3.** Pairwise comparisons for the number of shared features.

Contrast (features)	Estimate (log ms)	*SE*	*M*_diff_ (ms)	*z*	*p*	*d*
0 vs. 1	−0.024	0.003	−5	−6.98	<0.001	−0.14
0 vs. 2	−0.076	0.004	−16	−17.68	<0.001	−0.44
0 vs. 3	−0.157	0.005	−36	−34.05	<0.001	−0.90
1 vs. 2	−0.052	0.002	−11	−34.05	<0.001	−0.30
1 vs. 3	−0.133	0.004	−31	−30.38	<0.001	−0.77
2 vs. 3	−0.081	0.003	−20	−23.37	<0.001	−0.47

*Note:* *M*_diff_ = Numbers 0–3 denote the number of shared features in each condition. Mean difference between number of shared features in milliseconds.

### Feature type

Whereas the feature-count analysis establishes that disengagement costs scale nonlinearly with overlap, the following component decomposition provides the more informative test: It reveals whether this nonlinearity reflects overlap count per se or specific feature combinations. To examine the independent and combined effects of the different shared features on SL, we conducted a component decomposition analysis including three binary predictors: Object category (O), Color (C), and Shape (S). Each predictor was coded 1 if the corresponding feature was shared between the center and target stimulus and 0 if it was not shared. The model included all main effects (O, C, S) and their interactions (C × O, C × S, O × S, and O × C × S), as well as the control variables block number, object-category set, and trial position. Random intercepts and random slopes for object-category set were estimated for participants. The results of the model can be found in Table 4.

**Table 4.** Type III ANOVA results for the component decomposition analysis.

Predictor	*df*_1_	*df*_2_	*F*	*p*	$\eta_{p}^{2}$
O	1	17,160.6	0.05	0.815	<0.001
C	1	17,163.2	153.80	<0.001	0.009
S	1	17,165.4	0.00	0.954	<0.001
O × C	1	17,163.0	14.03	<0.001	0.001
O × S	1	17,164.6	2.52	0.113	<0.001
C × S	1	17,163.0	83.06	<0.001	0.005
O × C × S	1	17,168.4	2.04	0.153	<0.001
Block number_*c*_	1	17,232.7	2.57	0.109	<0.001
Object-category set	3	38.1	3.11	0.038	0.197
Trial number_*c*_	1	17,172.3	22.09	<0.001	0.001
Block number_*c*_ × Object-category set	3	6,337.0	2.95	0.032	0.001

*Note:* O = object category; C = color; S = shape. Block number_*c*_ and trial number_*c*_ were mean-centered. Degrees of freedom are Satterthwaite approximations.

The analysis revealed a significant main effect of Color, indicating that shared color substantially increased saccadic latency. Neither the main effects of object category nor shape were significant. Significant two-way interactions emerged for color and object category (C × O) and color and shape (C × S), but not for object category and shape (O × S). The significant two-way interactions revealed nonadditive (super-additive) effects for color and shape (C × S) and color and object category (C × O), meaning that the effects were larger than one would expect when summing up the effects of the corresponding two main effects. The three-way interaction (O × C × S) was not significant, indicating that when all three features were shared, saccadic latency followed the pattern expected from the additive combination of the lower-order effects. The estimated means for each of the feature combinations can be found in Table 5, and a graphical depiction can be found in Figure 5. Again, saccadic latency decreased with trial number, reflecting within-block practice effects. There was a significant main effect for object-category set but no main effect for block number. The interaction between block number and object-category set was significant, suggesting that participants showed different learning trajectories across blocks depending on object-category set. The fixed effects explained 8.6% of the variance in log-transformed saccadic latency (marginal *R*^2^ = 0.09), whereas the full model including random effects explained 44.9% (conditional *R*^2^ = 0.45).

**Table 5.** Estimated marginal means of saccadic latency by feature combination.

O	C	S	*M* (ms)	*SE*	95% CI_*LL*_	95% CI_*UL*_
0	0	0	214	4.43	205	223
1	0	0	214	4.42	205	223
0	1	0	228	4.73	219	238
0	0	1	214	4.43	205	223
1	1	0	234	4.85	225	244
1	0	1	216	4.47	208	225
0	1	1	244	5.05	234	254
1	1	1	249	5.17	239	260

*Note:* O = object category; C = color; S = shape. 0 = feature not shared with the target; 1 = feature shared with the target.

## Discussion

The present study examined how distractor–target similarity influences *oculomotor disengagement* in visual search, operationalized as the latency to initiate the first saccade away from the center stimulus. Two complementary analyses converge on a clear pattern. First, saccadic latency increased with the *number* of shared features between the fixated center stimulus and the target, and this increase was better captured by a quadratic than by a linear function. Second, this nonlinear scaling was not a general consequence of “more overlap” across arbitrary dimensions; rather, it was driven primarily by *color* similarity and by pairwise interactions in which color amplified the impact of additional overlaps. In what follows, we integrate these results with priority-map accounts and two-stage frameworks of search and propose a mechanistic interpretation in terms of hierarchical gating into verification.

### Delayed-disengagement costs increase with feature overlap

At the level of feature count, the data show a robust delayed-disengagement effect: Saccadic latency increased monotonically from the zero-overlap baseline to the full-overlap condition. This extends classic observations that fixated, target-matching stimuli can hold gaze and delay leaving the current fixated stimulus (Boot & Brockmole, 2010; Blakely et al., 2012; Stefani et al., 2020; Saalwirth et al., 2024). Critically, because overlap was graded from zero to three shared dimensions, the present design allowed us to test the shape of this relationship. The quadratic model provided a better fit than a linear model, indicating that disengagement costs accelerated as overlap increased.

From the perspective of two-stage frameworks such as guided search (Wolfe, 2021), a convex increase is theoretically meaningful: Low-similarity stimuli can be rejected via a rapid feature-level check, whereas higher-similarity candidates are more likely to trigger capacity-limited verification before a saccade is initiated. In this sense, the quadratic trend is consistent with the idea that the system transitions from efficient rejection to time-consuming verification as overlap rises.

However, the feature-count analysis alone cannot identify which dimensions drive this nonlinearity, because the one-feature and two-feature levels aggregate heterogeneous combinations. The component decomposition analysis resolves this ambiguity and shows that the quadratic trend reflects which features overlap, not merely how many overlap.

### A hierarchical gating account

The component decomposition yields three key findings. First, color similarity produced a substantial increase in saccadic latency, whereas shape-only and object-category-only overlap produced latencies near baseline. Second, the strongest departures from additivity were *two-way* interactions involving color: Color × shape and color × object category produced larger delays than expected from summing their single-feature contributions. Third, the absence of a three-way interaction indicates that the full-overlap condition does not require an additional, qualitatively distinct synergy beyond these lower-order effects.

This structure explains why a quadratic function fit the feature-count relationship better than a linear function. The one-feature level averages across a strong color-only cost and two near-zero costs (shape-only and object-category-only). By contrast, the two-feature level includes two color-containing combinations that are amplified by over-additive interactions (C × S; C × O). As a consequence, the increase from one to two shared features is larger than the increase from zero to one, yielding an apparent acceleration in saccadic latency when overlap is parameterized only by feature count. Thus, the nonlinearity is real, but it is best understood as emerging from conditional feature contributions rather than from a uniform “each additional feature adds the same cost” rule.

The pattern in the full-overlap condition further constrains interpretation. Because the three-way interaction was not significant, the latency when all three dimensions overlapped does not require an additional “all-features” penalty beyond the lower-order terms. Put differently, the full-overlap latency can be explained as the color-match latency plus two conditional increments: (a) the extra delay produced by adding object-category overlap when color already matches (i.e., the O × C – C difference) and (b) the extra delay produced by adding shape overlap when color already matches (i.e., the C × S – C difference). Empirically, these two increments closely reconstruct the full-overlap mean: Color × object category (234 ms) exceeds color (228 ms) by about 6 ms, color × shape (244 ms) exceeds color by about 16 ms, and adding these increments to the color baseline predicts roughly 250 ms, matching the observed color × shape × object category latency of about 249 ms. This indicates that the highest latencies in the full-match condition are fully accounted for by color and its pairwise interactions with object category and shape, without additional three-way synergy. We note, however, that the study was powered for the single-feature color-overlap contrast rather than for these interaction terms; the interaction estimates underlying this decomposition are therefore best read as effect sizes to be confirmed in adequately powered replications.

Taken together, the results are well captured by a candidate hierarchical gating process during fixation, which we advance as a promising interpretation rather than an established mechanism. In this account, disengagement begins with a fast evaluation of highly weighted, rapidly available information. Color is an especially plausible candidate for this role because it is typically extracted quickly and provides a highly diagnostic match signal (Wolfe, 2021). If the fixated stimulus mismatches on this gate dimension, it is treated as a low-probability candidate and can be rejected with minimal additional scrutiny, yielding near-baseline SL even when other properties (shape or object category) overlap. If the fixated stimulus matches on color, candidate probability increases and a slower verification process is engaged; within this verification mode, additional overlap dimensions (shape and object category information) prolong the time required to reach a confident rejection decision, delaying saccade initiation. This asymmetry is theoretically meaningful rather than incidental: As established in the Introduction, object category—unlike color or shape—is not a single visual feature but a higher-level, template-based representation, so its influence is expected to depend on co-occurring low-level overlap. That object-category overlap mattered only once a color match had engaged verification is therefore consistent with a gating view rather than anomalous, a point we take up in more detail below.

This interpretation naturally produces the observed over-additive interactions. Under gating, shape and object-category overlap do not contribute independently at all times; they matter primarily when the system has already committed to verification because the gate has been passed. That is, color overlap changes the processing regime, and within that regime, additional overlap increases verification time. The absence of a three-way interaction is also expected: Once verification is engaged, adding another matching dimension can extend verification, but it need not introduce an additional, qualitatively new stage beyond the verification process already underway.

The present results therefore refine the general distinction between oculomotor capture and disengagement (Born et al., 2011; Stefani & Sauter, 2023). Whereas capture determines which stimulus initially wins competition, the current data emphasize that disengagement depends on whether the fixated stimulus is rapidly rejected or treated as a plausible candidate requiring further verification. Color appears to be the strongest determinant of this stay-versus-switch decision.

We emphasize, however, that the present design does not uniquely adjudicate between this gating account and a simpler priority-weighting account, in which color is not a discrete gate but merely receives a substantially higher weight than shape or category—whether because it was more salient, more discriminable, or strategically more useful for identifying the target. Both accounts predict the observed color-dominated interaction pattern, and the two are not mutually exclusive. We therefore frame hierarchical gating as consistent with, rather than established by, the present data. A further reason for caution is that the three feature dimensions were not equated for discriminability: Color’s privileged role may thus reflect characteristics of the present stimulus set and task rather than a general property of oculomotor disengagement. Adjudicating between these accounts—and establishing the generality of color’s privileged role—would require designs that equate dimensions for discriminability or that omit color entirely.

### Why did shape and object-category overlap not show independent effects?

The near-zero costs of shape-only and object-category-only overlap do not imply that these dimensions are never processed. Rather, they suggest that, in the time window that determines first-saccade initiation, shape and object-category information were not sufficiently diagnostic to delay the leave decision unless color already indicated target similarity. This is compatible with previous findings that color often dominates early control signals and that shape effects become pronounced when shape discriminability or salience is experimentally enhanced (Hao et al., 2022). In the present object-category sets, shape may have been less discriminable than color for immediate rejection decisions, leading to a strategy in which shape alone rarely triggers extended verification.

For object-category overlap, a similar argument applies, but for different reasons. Category is not a primitive feature; it is a composite property inferred from multiple visual cues and tends to guide selection most strongly when category structure is visually coherent and template formation is feasible (Hout et al., 2017; Robbins & Evdokimov, 2024). In addition, evidence from functional identity paradigms suggests that identity-level evaluation is often gated by feature overlap (Wright et al., 2015; Stefani et al., 2020). The present finding that object-category overlap mainly mattered in color-containing combinations fits this broader pattern: Higher-level similarity may contribute primarily during verification, not as a standalone driver of the initial disengagement decision.

### Template precision and category structure as moderators of verification costs

The color-dominated gating pattern also provides a concrete way to link the results to template-precision accounts. If observers can maintain a precise template for a highly reliable dimension, mismatches can be rejected quickly, while matches become strong candidates that demand verification. This trade-off aligns with the broader literature showing that precise templates can improve guidance yet prolong verification in target-like clutter, especially under high within-category similarity (Hout et al., 2017; Robbins & Evdokimov, 2024). In the current data, a short saccadic latency in noncolor conditions is consistent with efficient rejection under a strong color-based template, whereas amplified costs for color-containing overlaps indicate that the same template increases the probability that verification is engaged and extended.

At the same time, the weak standalone object-category effects suggest that the category representation available in this task may have been comparatively coarse at the moment of disengagement. If within-category variability was substantial, or if category-consistent distractors were not visually coherent enough to support a precise category template, category overlap would be expected to exert limited control over early saccade initiation, while still contributing once verification is triggered by a strong low-level match.

Treating object-category overlap as a binary feature is experimentally convenient, but conceptually, it is unlikely to reflect a single, elementary dimension in the same sense as color. Object-category overlap plausibly bundles multiple cues (shape components, texture, parts, and semantic associations) and may reflect a category-level match rather than identity-level sameness. The present interaction pattern suggests that whatever information was captured by the “object category” factor influenced disengagement mainly *after* a strong candidate signal (color match) had already engaged verification. This distinction matters for interpretation: A weak main effect does not mean “no object-category processing,” but rather that object-category information was not the primary determinant of whether verification was entered in the first place. Conversely, because within-category exemplars necessarily shared some residual low-level similarity with the target, part of the C × O interaction may reflect this retained low-level overlap rather than category-level processing alone. The present design cannot fully separate these two contributions, and we therefore interpret the category-related effects as an upper bound on genuine category-level influence.

Thus, an additional possibility is that color received such a high weight in the priority policy of the present task that it dominated the disengagement decision and effectively reduced the contribution of other dimensions in the critical time window. Under this dominance account, the near-zero effects of shape-only and object-category-only overlap should be interpreted cautiously: They may reflect not only limited discriminability or slower availability of these dimensions, but also that their potential influence was masked by the strong reliance on color as the primary match cue. Further experiments would therefore have to omit color as a dimension of features and investigate whether shape and object-category overlap then cause measurable costs for the separation of individual features. Only then can one conclude with confidence that the one-feature effects for shape and object category are genuinely absent rather than suppressed by color dominance.

One further limitation concerns stimulus validation. Because the critical stimuli were AI-generated and the experimental logic rests on the intended similarity relations among them, the correspondence between the designed overlap structure and participants’ perceived similarity was not independently validated. The systematic, condition-dependent latency pattern indicates that the manipulations were effective, but future work could verify the intended color, shape, and category relations with explicit similarity ratings.

### Summary

In summary, disengagement costs increased with the number of shared features and showed a nonlinear (quadratic) scaling at the level of feature count. This nonlinearity is most parsimoniously described in terms of a color-dominated processing regime, which we interpret as a candidate gating architecture: Color overlap determines whether the fixated stimulus is rejected rapidly or enters a verification mode, and within that verification mode, additional overlap in shape or object-category information produces over-additive delays. The absence of a three-way interaction indicates that full-overlap costs are fully accounted for by these lower-order, color-dependent components, constraining models of how similarity governs the temporal dynamics of attentional selection.
